# Supplementary material for: The genome sequence of the Bronze Shieldbug, Troilus luridus (Fabricius, 1775)
Source: Wellcome Open Res. 2025 May 20;10:259. [Version 1] doi: 10.12688/wellcomeopenres.24260.1 (PMC13173287; doi:10.12688/wellcomeopenres.24260.1)
Supplement: Peer review history

# Reviewer report: Reviewer response for version 1

**DOI:** 10.21956/wellcomeopenres.26757.r153625

**Published:** 2026-05-13

**Authors:** Annabel Whibley

**Affiliations:** 1 The University of Auckland, Auckland, New Zealand

In this Data Note, Crowley and colleagues report the genome assembly of the Hemipteran Bronze Shieldbug, *Troilus luridus*. Sample identity was verified by barcoding and PacBio HiFi (Revio and Sequel IIe) and HiC data contributed to the assembly. The methods and reporting follow DToL templating and are well-structured and comprehensive, with public accession links and extensive analysis and metadata to support the excellent quality of the resulting assembly.

 I note that there is considerable sub-structure to the HiC heatmap, but the diagonal signal remains strong and so I interpret this as interactions within chromosome arms rather than misassembly. The BlobPlot shows a few clouds of small scaffolds, including a cluster with high GC content, reflecting the challenges of assembling a relatively large and heterozygous genome. Nevertheless, >93% of the assembly was scaffolded to haplotypes and completeness and contiguity stats are excellent for the taxon.

 The assembly is presented as two haplotypes- one curated to chromosome level and containing both sex chromosomes, the other curated just to scaffold level. Whilst this is a practical solution- a useful reference for population studies would indeed include X and Y- I did also find it triggered a bit of a semantic debate about what constitutes a haplotype. I wonder whether there might be  different term or qualifier that should be applied for a haplotype which is not, strictly, a “real” biological unit but nevertheless represents a single copy of each chromosome within a genome.

Are sufficient details of methods and materials provided to allow replication by others?

Yes

Is the rationale for creating the dataset(s) clearly described?

Yes

Are the datasets clearly presented in a useable and accessible format?

Yes

Are the protocols appropriate and is the work technically sound?

Yes

Reviewer Expertise:

Genomics, Bioinformatics

I confirm that I have read this submission and believe that I have an appropriate level of expertise to confirm that it is of an acceptable scientific standard.

# Reviewer report: Reviewer response for version 1

**DOI:** 10.21956/wellcomeopenres.26757.r149489

**Published:** 2026-03-19

**Authors:** Sivasankaran Kuppusamy

**Affiliations:** 1 Loyola College, Chennai, Tamil Nadu, India

The chromosome-level genome sequence of *Troilus luridus* (Fabricius, 1775) has been generated by the authors. Using whole-genome sequencing data, the authors assembled two haplotypes with total lengths of 1,044.50 Mb and 842.58 Mb, respectively. The haplotypes were further scaffolded into chromosomal pseudomolecules. Appropriate methodologies appear to have been used for sequencing, genome assembly, and annotation.

**Comments on the manuscript:**

 In the Abstract, the authors state: “Most of haplotype 1 (93.48%) is scaffolded into 8 chromosomal pseudomolecules, including the X and Y sex chromosomes. Most of haplotype 2 (93.27%) is scaffolded into 6 chromosomal pseudomolecules.” However, in the Assembly Statistics section, it is mentioned that “haplotype 2 was assembled to scaffold level.” This appears inconsistent with the statement in the Abstract. The authors are requested to verify and correct this discrepancy.

 The full genus name *Troilus luridus* has been used throughout the manuscript. According to standard scientific writing conventions, the full name may be used at the first mention, after which it should be abbreviated as *T. luridus* in subsequent occurrences.

 Overall, the research article is well prepared, and the manuscript meets the necessary scientific standards. It is suitable for indexing.

Are sufficient details of methods and materials provided to allow replication by others?

Yes

Is the rationale for creating the dataset(s) clearly described?

Yes

Are the datasets clearly presented in a useable and accessible format?

Yes

Are the protocols appropriate and is the work technically sound?

Yes

Reviewer Expertise:

Phylogenetic analysis of superfamily Noctuoidea moths using mitogenome sequences

I confirm that I have read this submission and believe that I have an appropriate level of expertise to confirm that it is of an acceptable scientific standard.
